# Effect of Anxiety About COVID-19 Infection in the Workplace on the Association Between Job Demands and Psychological Distress

**DOI:** 10.3389/fpubh.2021.722071

**Published:** 2021-10-13

**Authors:** Hisashi Eguchi, Ayako Hino, Akiomi Inoue, Mayumi Tsuji, Seiichiro Tateishi, Hajime Ando, Tomohisa Nagata, Shinya Matsuda, Yoshihisa Fujino

**Affiliations:** ^1^Department of Mental Health, Institute of Industrial Ecological Sciences, University of Occupational and Environmental Health, Japan, Kitakyushu, Japan; ^2^Institutional Research Center, University of Occupational and Environmental Health, Japan, Kitakyushu, Japan; ^3^Department of Environmental Health, School of Medicine, University of Occupational and Environmental Health, Japan, Kitakyushu, Japan; ^4^Department of Occupational Medicine, School of Medicine, University of Occupational and Environmental Health, Japan, Kitakyushu, Japan; ^5^Department of Work Systems and Health, Institute of Industrial Ecological Sciences, University of Occupational and Environmental Health, Japan, Kitakyushu, Japan; ^6^Department of Occupational Health Practice and Management, Institute of Industrial Ecological Sciences, University of Occupational and Environmental Health, Japan, Kitakyushu, Japan; ^7^Department of Preventive Medicine and Community Health, School of Medicine, University of Occupational and Environmental Health, Japan, Kitakyushu, Japan; ^8^Department of Environmental Epidemiology, Institute of Industrial Ecological Sciences, University of Occupational and Environmental Health, Japan, Kitakyushu, Japan

**Keywords:** anxiety, COVID-19, Japan, psychosocial factors, psychological distress, workplace

## Abstract

**Purpose:** There is limited information about the association between workplace psychosocial factors and general worker mental health status during the COVID-19 pandemic. In the present study, we examined how anxiety about being infected by COVID-19 in the workplace affected the association between job demands and psychological distress (PD).

**Method:** A cross-sectional online survey was conducted in December 2020. The final analyzed sample was 27,036. The dependent variable of PD was assessed using the Kessler Psychological Distress Scale (K6). Job demands were assessed using the Job Content Questionnaire. Feelings of anxiety were assessed by participants' responses to the following question: “Do you feel anxiety about being infected by COVID-19 in the workplace?” We used a two-level regression adjusting for prefectural level: each individual-level variable at level 1 was nested into each prefecture at level 2, stratified by presence of anxiety.

**Results:** A total of 50.5% of participants felt anxious about being infected by COVID-19 in the workplace. The interaction between anxiety and job demands was significant. Job demands were positively associated with PD. In the stratified analysis, the associations were stronger among employees who experienced anxiety about COVID-19 infection in the workplace than among those who did not.

**Conclusion:** The association between job demands and PD may be strengthened by anxiety about COVID-19 infection in the workplace.

## Introduction

It has been widely demonstrated that work environment, organization, and work-related behaviors can affect the mental health and psychological well-being of workers (1, 2). These organizational factors may be affected by the current coronavirus disease 2019 (COVID-19) pandemic, with resulting exacerbation or moderation of mental health outcomes.

In Japan, the number of people who died of suicide increased from 20,169 in 2019 to 20,919 in 2020 (3, 4). High job demands cause depression, which can lead to suicide. Karoshi, or death from overwork, represents a growing public health issue in East Asia (5). Contact with death/suffering, fear of contagion, and workload have been identified as important job demands related to COVID-19, and have been treated by health professionals in different health contexts during the pandemic (6). However, research has mainly focused on health care workers, infected patients, and essential workers (6–12). Research on general workers is scarce (13, 14). Therefore, there is limited information about the association between job demands and general worker psychological distress during the third wave of the COVID-19 pandemic.

Anxiety about possible COVID-19 infection in the workplace is an important issue in occupational mental health (1). A previous study conducted during the first COVID-19 wave demonstrated a positive association between the number of infection-control measures and anxiety about COVID-19 (15). Changes in worker anxiety may be affected by the effects of the COVID-19 pandemic on workplaces. A previous study showed that low personal resources, a factor identified as a predictor of anxiety (16), strengthens the association between job demands and psychological distress (17). Many people express distrust and anger toward others because of anxiety about COVID-19. Anxiety about COVID-19 may affect the association between job demands and psychological distress during the COVID-19 pandemic. However, most previous studies were conducted in the first and second COVID-19 waves and there is little information on the effect of COVID-19-related anxiety on worker mental health during the third COVID-19 wave.

In a previous UK study, anxiety levels were highest at the start of lockdown and showed improvement over time (18). In contrast, some Japanese studies have identified a deterioration in employee mental health following repeated COVID-19 outbreaks (19, 20). The third wave of COVID-19 infections in Japan began in December 2020. Our survey was launched on December 22, 2020. By December 26, there were new record highs for COVID-19 infections, deaths related to the disease, and the number of severe cases, just before the government declared a second state of emergency in the greater Tokyo area on January 7, 2021, almost 1 year since the beginning of the COVID-19 pandemic. This state of emergency was expanded to seven prefectures on January 13. The third COVID-19 wave may have had different effects on worker mental health than the first and second waves. A fourth state of emergency was declared on July 12, 2021, because of a fifth wave of COVID-19 infection. Compared with previous waves, the fifth wave has been characterized by a higher number of daily cases, more infections among middle-aged and younger people (including those of working age), and the threat of the more severe Delta variant, but far fewer deaths and patients with severe symptoms requiring intensive care. Most adults with COVID-19 in Japan can discontinue isolation and precautions 10 days after symptom onset, if fever has resolved for at least 72 h (without the use of fever-reducing medications) and if other symptoms have improved.

**Figure 1:**
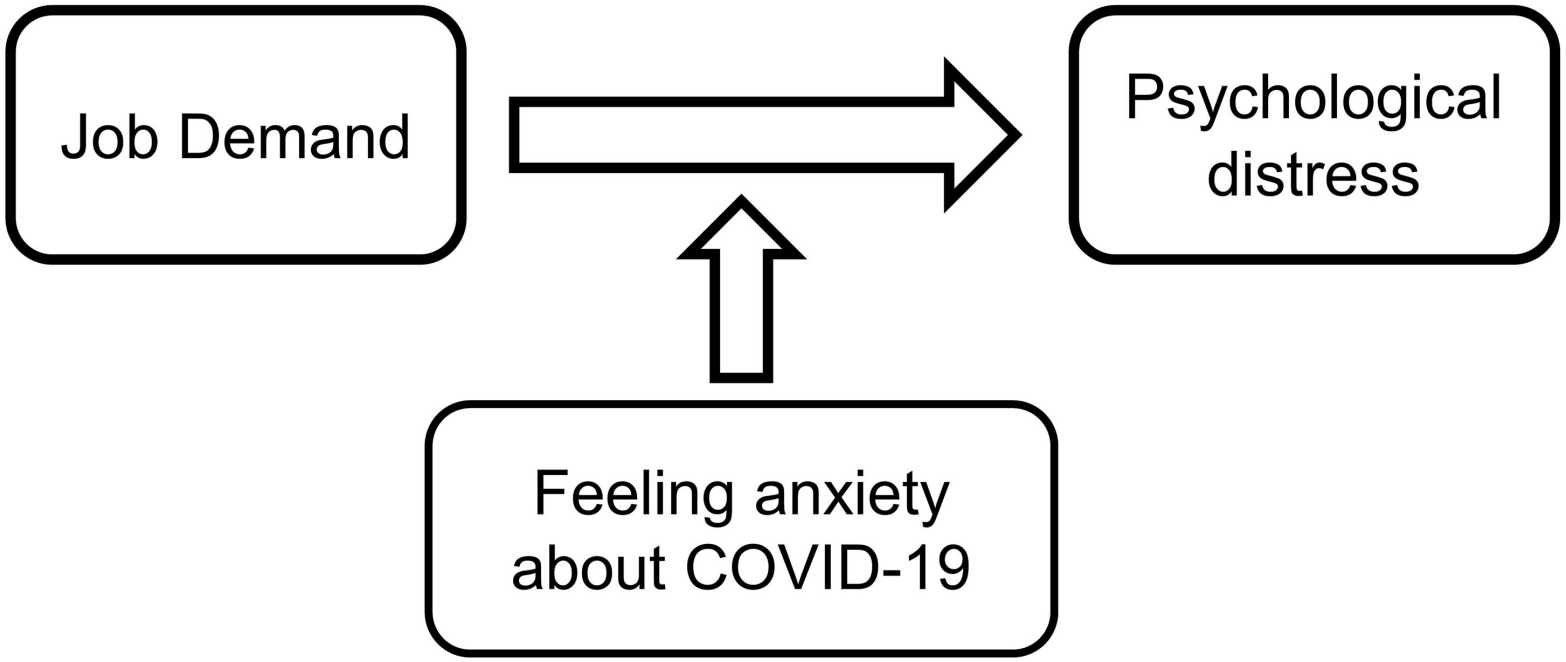
Conceptual model of possible associations between felt anxiety about COVID-19, job demand, and psychological distress.

In the present study, we hypothesized that anxiety about being infected by COVID-19 in the workplace may strengthen the association between higher job demands and worse psychological distress among general Japanese workers during the third wave of COVID-19 infections. Figure 1 shows the concept model underlying the study hypothesis.

## Materials and Methods

A cross-sectional online survey was conducted in December 2020 among participants who had previously registered with a Japanese web survey company. Invitations to participate were sent to 665,381 registrants via e-mail. Details of the survey protocol have already been reported (21). A sampling plan was designed to recruit an equal number of respondents from 20 collection units, each comprising a combination of five regions, with comparable sex and office/non-office worker status. The target sample size was 30,000, 1,500 respondents from each collection unit. A total of 1,650 respondents (the target sample size plus a margin of 10%) were recruited from each collection unit. Of the 33,302 eligible respondents, 215 were excluded because they were deemed to have provided fraudulent responses by Cross Marketing Inc., leaving 33,087 respondents. Participants were selected using a random number generator. The study population comprised individuals interested in participating in a survey. There was a modest financial incentive for survey participation (equivalent to a few US dollars). Subsequently, 6,051 surveys with invalid responses or response errors were excluded, leaving 27,036 surveys for inclusion in the study. The exclusion criteria were as follows: extremely short response time ( ≤ 6 min), extremely low body weight (<30 kg), extremely short height (<140 cm), inconsistent answers to similar questions throughout the survey (e.g., inconsistent responses to questions about marital status and living area), and incorrect answers to a staged question used to identify fraudulent responses (“Choose the third-largest number from the following five numbers”).

The study aims and protocol were approved by the ethics committee of the University of Occupational and Environmental Health, Japan (R2-079). Informed consent to participate in this study was obtained from all participants. Participants were informed in advance that their participation was strictly voluntary and that all information they provided would remain confidential. Individuals who consented to participate were able to access a designated website (after confirmation of their personal information) on which they could complete the survey. Participants had the option to not respond to any part of the questionnaire, and could discontinue participation at any time.

### Dependent Variable: Psychological Distress

Psychological distress was assessed using the Kessler Psychological Distress Scale (K6). The K6 was originally developed as a screening instrument for non-specific psychological distress and serious mental illness. Its internal reliability and validity have been documented (22). The K6 consists of a six-item battery asking how frequently respondents have experienced symptoms of psychological distress in the past 30 days. Responses range from 0 (none of the time) to 4 (all of the time); total scores range from 0 to 24. The K6 has been translated into Japanese, and the Japanese version has been validated. In this sample, Cronbach's α coefficient for the K6 was 0.88.

### Independent Variable: Job Demands

We used the job demands scale from the Japanese version of the Job Content Questionnaire (JCQ) (23). The JCQ, developed by Karasek, is based on the job demands–control (or demand–control–support) model and contains five items to assess job demands (five items), rated on a 4-point scale (1 = strongly disagree to 4 = strongly agree). The reliability and validity of the Japanese version of the JCQ are acceptable, as shown by Kawakami et al. (23). In the present study, Cronbach's α coefficient for job demands was 0.68.

### Moderator Variable: Feeling Anxiety About COVID-19

Anxiety about being infected by COVID-19 was determined by participants' responses to the following question: “Do you feel anxiety about being infected by COVID-19 in the workplace?” Responses were dichotomized on a two-point scale: 1 = yes and 0 = no.

### Assessment of Covariates

Covariates were measured using a self-administered questionnaire, and included demographic and lifestyle characteristics such as sex, age, marriage, educational attainment, occupation, job type, and annual family income. Age was expressed as a continuous variable. Marriage was classified into three categories: married, divorced/widowed, and not married. Educational attainment was classified into three categories: junior high school and high school, college, and technical school, and university and graduate school. Occupation was classified into 10 categories: staff member; manager; executive; public official/teaching staff/non-profit organization employee; temporary and contract employee; self-employed person; small office/home office worker; agriculture, forestry, and fishery worker; professional (e.g., lawyer, accountant, medical doctor); and others. Job type was classified into three categories: mainly desk work (clerical or computer work), mainly talking to people (e.g., customer service, sales, selling), and mainly labor (e.g., work at construction sites, physical work, nursing care). Company size was categorized into 10 groups: 1 (self-employment), 2–4, 5–9, 10–29, 30–49, 50–99, 100–499, 500–999, 1,000–9,999, and 10,000 or more. Participants were asked to indicate their yearly equivalent household income by choosing one of five income bands: (i) 47.4–224.5 ten thousand JPY; (ii) 225.0 to 317.5 ten thousand JPY; (iii) 318.1 to 428.7 ten thousand JPY; (iv) 433.0 to 525.0 ten thousand JPY; (v) 530.3 to 1050.0 ten thousand JPY. The presence of chronic disease was determined by participants' responses to the following question: “Do you have any diseases that require regular visits to the hospital or treatment?” Responses were dichotomized on a two-point scale: 1 = yes and 0 = no. We categorized participants who chose the following disease as having chronic disease; diabetes, high blood pressure, angina/myocardial infarction, depression or other mental illness, asthma and other lung diseases, otolaryngological disorders, skin disease, low back pain and other joint diseases, gynecological disorders, infertility, cancer, gingival therapy, and others. In addition, the cumulative incidence rate of COVID-19 infection 1 week prior to the survey in the residential prefectures was used as a prefecture-level variable. This information was collected from the websites of public institutions.

### Statistical Analysis

Analysis of variance and the chi-square test were conducted to examine differences in demographic variables and psychological distress between participants who were anxious about COVID-19 infection and those who were not. We used multilevel regression with two levels adjusting for prefectural level: each individual-level variable at level 1 was nested into each prefecture at level 2. We examined the interaction between COVID-19 anxiety and job demands. We found a significant interaction between COVID-19 anxiety and job demands (*p* = 0.012). To compare the adjusted coefficients between presence or absence of COVID-19 anxiety in the workplace, multiple regression analyses were used to examine the association between job demands and psychological distress stratified by anxiety about COVID-19 in the workplace. We conducted multiple regression analysis using a crude model (Model 1) and a model adjusted for sex, age, marital status, educational attainment, occupation, job type, weekly working hours, annual household income, company size, and chronic disease (Model 2).

All analyses were performed using Stata 15SE (StataCorp, College Station, TX, USA), with statistical significance set at p < 0.05.

## Results

Approximately half of participants answered yes to the question “Do you feel anxiety about being infected by COVID-19 in the workplace?” Employees who felt anxious about being infected by COVID-19 in the workplace were younger and had greater psychological distress. Women, staff members, workers whose job mainly involved talking to people, employees in larger companies, and those with no chronic disease were more likely to feel anxious about workplace COVID-19 infection (Table 1).

**Table 1:** Participant characteristics (*n* = 27,036).

	**Do you feel anxiety about being infected by Covid-19 in the workplace?**				
	**Yes (*****n*** **=** **13,646)**		**No (*****n*** **=** **13,390)**		***p***
Age (years) (SD)	45.8	(10.7)	48.2	(10.2)	<0.001^a^
Range (minimum–maximum)	20–65		20–65		
Job demands (job content questionnaire) (SD)	31.0	(6.0)	29.1	(5.6)	<0.001^a^
Range (minimum–maximum)	12–48		12–48		
Psychological distress (K6) (SD)	5.5	(5.7)	3.8	(5.0)	<0.001^a^
Range (minimum–maximum)	0–24		0–24		
**Sex**					
Men	5,952	(43.6)	7,862	(58.7)	<0.001^b^
Women	7,694	(56.4)	5,528	(41.3)	
**Marital status**					
Married	7,468	(54.7)	7,561	(56.5)	0.014^b^
Divorced or widowed	1,450	(10.6)	1,393	(10.4)	
Unmarried	4,728	(34.7)	4,436	(33.1)	
**Educational attainment**, ***N*** **(%)**					
Junior high school and high school	3,500	(25.7)	3,821	(28.5)	<0.001^b^
College and technical school	3,616	(26.5)	2,928	(21.9)	
University and graduate school	6,530	(47.9)	6,641	(49.6)	
**Occupation**, ***N*** **(%)**					
Staff member	6,760	(49.5)	5,815	(43.4)	<0.001^b^
Manager	1,203	(8.8)	1,338	(10.0)	
Executive	254	(1.9)	608	(4.5)	
Public official, teaching staff, or non-profit organization employee	1,398	(10.2)	1,412	(10.6)	
Temporary and contract employee	1,651	(12.1)	1,243	(9.3)	
Self-employed person	742	(5.4)	1,487	(11.1)	
Small office/home office worker	75	(0.6)	303	(2.3)	
Agriculture, forestry, and fishery worker	24	(0.2)	188	(1.4)	
Professional (e.g., lawyer, accountant, medical doctor)	1,281	(9.4)	566	(4.2)	
Other	258	(1.9)	430	(3.2)	
**Job type**					
Mainly desk work (clerical or computer work)	6,182	(45.3)	7,286	(54.4)	<0.001^b^
Mainly talking to people (e.g., customer service, sales)	4,049	(29.7)	2,878	(21.5)	
Mainly labor (e.g., work at construction sites, physical work, nursing care)	3,415	(25.0)	3,226	(24.1)	
**Yearly household income (ten thousand JPY)**, ***N*** **(%)**					
1 (47.4–224.5)	2,446	(17.9)	2,657	(19.8)	<0.001^b^
2 (225.0–317.5)	2,969	(21.8)	2,643	(19.7)	
3 (318.1–428.7)	2,709	(19.9)	2,552	(19.1)	
4 (433.0–525.0)	2,559	(18.8)	2,474	(18.5)	
5 (530.3–1050.0)	2,963	(21.7)	3,064	(22.9)	
**Company size**, ***N*** **(%)**					
1 (Self-employment)	705	(5.2)	1,851	(13.8)	<0.001^b^
2–4	742	(5.4)	1,254	(9.4)	
5–9	769	(5.6)	844	(6.3)	
10–29	1,427	(10.5)	1,371	(10.2)	
30–49	830	(6.1)	762	(5.7)	
50–99	1,419	(10.4)	1,131	(8.5)	
100–499	2,892	(21.2)	2,264	(16.9)	
500–999	1,150	(8.4)	847	(6.3)	
1,000–9,999	2,587	(19.0)	2,132	(15.9)	
≥10,000	1,125	(8.2)	934	(7.0)	
**Chronic disease**					
Yes	8,510	(62.4)	9,016	(67.3)	<0.001^b^
No	5,136	(37.6)	4,374	(32.7)	

a *Analysis of variance*,

b *chi-square test. SD, standard deviation*.

We demonstrated a significant association between job demands and psychological distress, and also between anxiety about COVID-19 and job demands (*p* < 0.05). The stratified analysis (*post-hoc* simple slope analysis) showed that the effect of job demands was stronger among employees who felt anxious about COVID-19 infection in the workplace (coefficient = 0.16, yes = 1) than among those who did not (coefficient = 0.14, no = 0) (Table 2).

**Table 2:** Associations between job demands and K6 score by anxiety about workplace COVID-19 infection (*n* = 27,036).

**Anxiety about COVID-19 in the workplace**	**Model 1**		**Model 2**	
**Yes (*n* = 13,646)**	**Coefficient**	**95% CI**	**Coefficient**	**95% CI**
Job demands	0.16	(0.14, 0.17)	0.16	(0.14, 0.18)
Sex	0.84	(0.65, 1.03)	−0.07	(−0.28, 0.15)
Age	−0.08	(−0.08, −0.07)	−0.07	(−0.08, −0.06)
Marital status	0.86	(0.75, 0.96)	0.53	(0.43, 0.64)
Educational attainment	−0.15	(−0.27, −0.36)	−0.07	(−0.19, 0.04)
Occupation	−0.04	(−0.08, −0.08)	−0.06	(−0.09, −0.02)
Job type	0.06	(−0.06, 0.17)	−0.21	(−0.33, −0.09)
Yearly household income	−0.49	(−0.56, −0.42)	−0.41	(−0.48,−0.34)
Company size	−0.03	(−0.07, 0.01)	−0.05	(−0.09, −0.02)
Presence of chronic disease	1.88	(1.69, 2.07)	2.16	(1.97, 2.35)
**No (n** **=** **13,390)**				
Job demands	0.14	(0.13, 0.16)	0.14	(0.12, −0.15)
Sex	1.00	(0.83, 1.17)	0.16	(−0.02, 0.35)
Age	−0.09	(−0.10, −0.08)	−0.08	(−0.09, −0.07)
Marital status	0.81	(0.72, 0.91)	0.47	(0.38, 0.57)
Educational attainment	−0.18	(−0.28, −0.08)	−0.09	(−0.19, 0.06)
Occupation	0.01	(−0.02, −0.04)	0.03	(−0.01, −0.06)
Job type	0.14	(0.03, 0.24)	−0.13	(−0.23, −0.02)
Yearly household income	−0.40	(−0.46, −0.34)	−0.32	(−0.38, −0.26)
Company size	0.00	(−0.03, 0.03)	−0.02	(−0.05, 0.01)
Presence of chronic disease	1.17	(1.00, 1.35)	1.64	(1.46, 1.81)

*K6, Kessler Psychological Distress Scale; CI, confidence interval*.

## Discussion

We conducted a large online survey on December 22, 2020, just before the government declared a second state of emergency in the greater Tokyo area on January 7, 2021. Women, younger workers, and those with jobs that mainly involved talking to people (e.g., customer service, sales) were more likely to feel anxious about COVID-19 infection in the workplace. The association between job demands and psychological distress was stronger among employees who felt anxious about being infected by COVID-19 in the workplace than among those who did not.

The strength of the association between job demands and psychological distress was the same as that found in a previous study (24). Anxiety about COVID-19 infection in the workplace strengthened the association between job demands and psychological distress. Individual differences in coping styles are associated with psychological vulnerability to stress. Variability in how persons respond to threats has consistently been identified as a risk factor for the development of mood and anxiety disorders (25). Anxiety about COVID-19 infection in the workplace may affect the individual coping styles and response to threats.

Considering previous findings that the number of workplace measures correlated positively with respondents' fear of and worry about COVID-19 (15), the quality of measures to reduce the fear of infection may be more important than the quantity of measures. Working from home may reduce the fear of infection at or on the way to work (26, 27). Furthermore, limiting the frequency and amount of access to television and web-based media to obtain information about COVID-19 may be effective in preventing anxiety about COVID-19 infection (28). Employers should consider research evidence and the opinions of health professionals when addressing employee anxiety about workplace COVID-19 infection.

The present findings showed that women, younger workers, and those in jobs involving talking to people may be vulnerable to anxiety about COVID-19 infection in the workplace. Some studies have considered the effect of the COVID-19 pandemic on mental health outcomes in vulnerable working populations (29, 30). Recent studies suggest that younger people or students experience a greater psychological effect of the outbreak and higher levels of depression (31, 32). In 2020, for the first time in 11 years, suicide rates in Japan increased. Most surprisingly, although suicides in men fell slightly, rates among women increased by nearly 15%. The present findings may help to explain these trends.

This study had some limitations. First, our study population required Internet access to complete the survey and therefore may have been more aware of COVID-19 infection through access to online information. People should be aware of the psychological risk of too much media exposure and control their access in health crises such as the COVID-19 breakout (28). Our results are not completely generalizable to individuals without Internet access or to those in other countries and settings. Second, further studies are needed to evaluate whether other confounding factors provide possible mechanisms for the observed attenuation in the associations between job demands, anxiety about COVID-19 infection in the workplace, and psychological distress. For example, we had no information about the personality traits of participants. In addition, we had no information about the number of confirmed cases in the workplace. Third, the study was cross-sectional, so no causal relationships could be determined. A further interventional or prospective study is needed to clarify potential causal associations between job demands, anxiety about COVID-19 infection in the workplace, and psychological distress in the Japanese working population. Fourth, anxiety about being infected by COVID-19 was evaluated using a single question with a binary response option; therefore, we were unable to evaluate the level and type of participants' anxiety. Finally, we should consider the possible effects of common method bias when interpreting the results.

## Conclusion

The association between job demands and psychological distress may be strengthened by anxiety about being infected by COVID-19 in the workplace.

## Data Availability Statement

The raw data supporting the conclusions of this article will be made available by the authors, without undue reservation.

## Ethics Statement

The studies involving human participants were reviewed and approved by this study was approved by the Ethics Committee of the University of Occupational and Environmental Health, Japan. Informed consent: Informed consent was obtained from all participants. Written informed consent for participation was not required for this study in accordance with the national legislation and the institutional requirements.

## The CORoNaWork Project

The current members of the CORoNaWork Project, in alphabetical order, are as follows: Dr Yoshihisa Fujino (present chairperson of the study group), Dr Akira Ogami, Dr Arisa Harada, Dr Ayako Hino, Dr Hajime Ando, Dr Hisashi Eguchi, Dr Kazunori Ikegami, Dr Kei Tokutsu, Dr Keiji Muramatsu, Dr Koji Mori, Dr Kosuke Mafune, Dr Kyoko Kitagawa, Dr Masako Nagata, Dr Mayumi Tsuji, Ms Ning Liu, Dr Rie Tanaka, Dr Ryutaro Matsugaki, Dr Seiichiro Tateishi, Dr Shinya Matsuda, Dr Tomohiro Ishimaru, and Dr Tomohisa Nagata. All members are affiliated with the University of Occupational and Environmental Health, Japan.

## Author Contributions

HE wrote the initial draft of the manuscript. AH, YF, MT, ST, HA, TN, AI, and SM contributed to the analyses and interpretation of the data and assisted in the preparation of the manuscript. HE, AH, YF, MT, ST, HA, TN, and SM contributed to the data collection. All authors critically reviewed the manuscript, approved the final version of the manuscript, agree to be accountable for all aspects of the work, and ensuring that questions related to the accuracy or integrity of any part of the work are appropriately investigated and resolved.

## Funding

This study was funded by a research grant from the University of Occupational and Environmental Health, Japan; a general incorporated foundation (Anshin Zaidan) for the development of educational materials on mental health measures for managers at small-sized enterprises; Health, Labour and Welfare Sciences Research Grants: Comprehensive Research for Women's Healthcare (H30-josei-ippan-002) and Research for the establishment of an occupational health system in times of disaster (H30-roudou-ippan-007); consigned research foundation (the Collabo-health Study Group); scholarship donations from Chugai Pharmaceutical Co., Ltd.; and the Japan Society for the Promotion of Science (JSPS KAKENHI: Grant Number JP20K10477).

## Conflict of Interest

The authors declare that this study received funding from a scholarship donation from Chugai Pharmaceutical Co., Ltd.

## Publisher's Note

All claims expressed in this article are solely those of the authors and do not necessarily represent those of their affiliated organizations, or those of the publisher, the editors and the reviewers. Any product that may be evaluated in this article, or claim that may be made by its manufacturer, is not guaranteed or endorsed by the publisher.
